# Malarial Hemoglobinuria

**Published:** 1908-03

**Authors:** E. F. Jones

**Affiliations:** Midville, Ga.


### MALARIAL HEMOGLOBINURIA.

BY E. F. JONES, M. D., MIDVILLE, GA.

The consideration of this subject involves so much of the general problem of malaria, that there is a constant temptation to depart from the lines marked out in such a discussion, and to go contrary to time and subject limitations. It also involves so much investigation into the functions of the liver, in the pathological processes due to malarial disease, that the writer frankly confesses his inability to deal with this subject as thoroughly as he would wish. His desire in this paper is to present a treatment for this malady that has proved very satisfactory to the physicians of his section and to reason out, as far as possible, the physiological principles on which this treatment is based. If, after doing this, his reasoning be found falacious, he will be amply repaid by any discussion this paper may provoke.

Malarial Hemoglobinuria is usually found in the regions where the Aestivo-autumnal parasite exists, and this organism is given by many authors as its sole cause. Others deny this, but admit their ignorance as to the causative agent, saying that it may be a micro-organism as yet undiscovered; that the cause is not the Aestivo-autumnal parasite alone, would seem to be proved by the fact that there are certain regions, such as the Roman Campagna, which have the severest forms of malarial fever, without the hemoglobinuria and others such as Sicily, in which it is present.

Dr. Charles Hicks, of Dublin, in a very strong paper read before the 1902 meeting of the Medical Association of Georgia, called attention to the influence of the belt of lime stone rock that runs diagionally across the Southern States, from the Atlantic into Texas, and defining sharply the red clay lands, covered with oak and hickory, from the light, sandy soil on which stand the forests of yellow pine with a matting of wire grass. It is in this red clay country, traversed by this stratum of rotten lime stone rock, that the disease in question flourishes. The writer practices on the extreme edge of this belt; to the East and North stretch the oak and hickory country of Burke, with its annual prevalence during the fall months, of hemoglobinuria; just across the Ogeechee, stretch the pine barrens of Emanuel, with intermittent and remittent fever in abundance, but no hemoglobinuria, except possibly here and there a sporadic case.

Poor drinking water, however, I believe to be only an auxiliary cause. In my opinion the disease is due to a general diathesis, superinduced by bad climate, bad drinking water, bad food and improper habits of life. It is the sum total of the effects upon the human system of causes just noted, aided and sustained by the influence of the blood destroying malarial parasite. The unfortunate victim of this diathesis has suspended above him, the Damocletion sword. The abused and long suffering organism hampered and harassed in trying to convert into healthy pabulum the morbid products of digestion, stands the strain as long as possible. A chill, caused by the sporulation of a crop of malarial parasites, severs the hair, the sword falls and the liver insulted and indignant at the excessive work thrown upon it, becomes a literal bile factory and proceeds to poison the system with its secretion. The free hemoglobin in the blood clogs up the kidneys in its efforts to escape and stimulates the liver to renewed morbid activity. To illustrate this action of the liver, let me quote from Landois: "Cholemia may, however, result from the excessive production of bile, which can not be completely discharged into the intestent and is thus reabsorbed. This takes place when the red corpuscles which furnish the material for the manufacture of the bile are destroyed in excessive amounts. From this material only can the liver elaborate bile. Under such circumstances a plug of inspissated secretion at times forms in the bile ducts, as a result of which in consequence of the stagnation of the bile, its re-absorption is in turn favored. The billiary acids in the blood dissolve the red corpuscles and this leads to the further formation of bile. The dissolved hemoglobin is transferred in a new bile pigment, while the globulin-body of the disintegrated hemoglobin may form casts in the renal tubules which are later washed into the urine.

This, to my mind, explains almost the entire phenomenal of malarial hemoglobinuria. The parasite plays an important part in the causation of the disease, but usually a minor part in the disease itself. Without its hematolytic action on the red cells, we would have no hemoglobinuria, but the disease being present, its toxins in themselves rarely endanger life. Through its action on the red cells, it sets free in the blood an excess of hemoglobin, this in turn causes an excessive elaboration of bile by the liver which because it can not be all discharged into the intestine, is absorbed into the blood. This again, disintegrates the red cells forming fresh hemoglobin, while the globunin bodies form casts, which block up the renal tubules, some of the hemoglobin escaping through the tubules and producing the characteristic hemoglobinuria. This, I am aware, does not explain the action of quinine in precipitating the disease, or aggravating it when it is present. Quinine is, however, a poison to the human organism, as well as to the malarial parasites. It is irritant to the kidney and may also be irritant to the liver. That it will produce the condition in those suffering from the diathesis, of which I have spoken, I am fully convinced, having seen it do so in several instances; this is, I think, more properly a quinine hematuria, which often disappears on the withdrawel of the quinine.

Malarial Hemoglobinuria is classified as one of the types of pernicious malaria; it is characterized by usually an initial chill, followed by high fever, excessive nausea and vomiting, prostration, a rapid weak pulse and profound jaundice. The most alarming symptom to the patient and often to the physician, is the hemoglobinuria. In the severe forms, the patient is in a profound state of toxemia and unless he receives skilled medical aid, soon succumbs. In the mild forms, there are few active symptoms of malaria, although a febrile condition may have been present for several days.

The prognosis is guardedly favorable, depending upon the severity of the symptoms, the strength of the patient and the degree of elimination by the bowels and kidneys.

In regard to the treatment of Malarial Hemoglobinuria, there is a wide diversity of opinion. The most mooted point is the exhibition of quinine. Hare in his Practical Therapeutics says: "In this form of pernicious malaria, quinine is to be used with caution, if at all." Anders recommends its use in small doses, but recites the fact that Plehn, Richardson and others claim that it is not only useless, but harmful. Wilcox, in his Treatment of Disease, just published, says: "Here, unless active parasites, are present in the blood, it is wise to omit quinine, for should they be found, the drug is strongly indicated." Forscheimer, in his Prophylaxsis and Treatment of Internal Diseases, 1906 Edition, says: "The question of giving quinine is much more complicated when one sees cases in which in successive attacks of malaria, even the smallest dose is followed by hemoglobinuria; here the physician must choose between two serious conditions and decide which of the two is more important to endure. As a rule, the whole question will be decided by the laiety for in black water regions there exists great prejudice against the use of quinine, a prejudice which it has been seen, is founded on facts."

The latter has been the experience of the writer. In the section in which he practices, let it be known by a physician that he uses quinine in this type of disease and his experience with it will usually terminate in short order. Nevertheless, with the system entirely clear by a vigorous process of elimination, the symptoms of malaria continuing, together with the finding of the parasite in the blood, there would seem to be no contra-indication to the guarded administration of quinine. However, owing to the prejudice against it, and the fact that in my experience direct anti-malarials have been rarely necessary, I have used it only a few times, relying on arsenic and other less active drugs. The treatment which the writer uses for this disease has been used with individual modifications in his locality for many years with signal success. Although it may be widely known, he has never seen it alluded to in any literature on this subject. In using it, he has merely followed the footsteps of his predecessors, and in presenting it, he lays no claim to bringing forward anything new, startling or original. The sole purpose of this paper is an effort to reason out the physiological principles on which the treatment is based. Taking the pathological view of the disease that he does, there are four cardinal therapeutic indications; first, to stimulate the liver and eliminate the poison as rapidly as possible; second, to keep the kidneys active; third, to maintain the strength of the patient, and fourth to ward off the subsequent chills which will throw more free hemoglobin in the blood. The first indication is met by giving calomel in large doses, ten to twenty grains every two hours till forty fifty or sixty grains are given followed by the saturated solution of Epsom Salts in broken doses in four hours after last dose of calomel, until excessively free catharsis is established. Usually after free elimination in this manner, the symptoms will subside and the urine become clear. If the jaundice or hemoglobinuria continue, other courses of calomel are indicated in slightly smaller doses until the jaundice is less profound and the hemoglobinuria disappears. No other drug will serve as well as calomel. In the language of a physician, long since gone to his reward, "Give all the calomel the system will stand—then twenty grains more."

You may, and often will, salivate your patient, but this can in most cases be avoided by the free use of Epsom Salts and a prophylactic mouth wash of Potassium Chlorate Tr. Myrrh, Listerine and water. The calomel acts, I believe, not as a direct chologogue, but by stimulating the duodenum causes the excessive bile to be thrown off through the bowels. It also acts as an intestinal antiseptic preventing the absorption of the poison in the intestines.

The second indication is not by refrigerant diuretics. Any one of Potassium Acetate in 20 to 30 grain doses every two to four hours, or Potassium Nitrate in 10 grain doses, or Potassium Birtartratein 20 to 30 grain doses may be employed until the kidney function is reestablished. These are often not well borne and in such cases, I have used a proprietory preparation called Nephroson or Sour-wood Diuretic with satisfaction. Cupping the kidneys hot fomentations, hot packs and entreoclysis freely are often of great service in re-establishing the renal secretion The third indication, of course, depends upon the vitality of the patient. As the heroic treatment given above is usually discontinued after the first 24 to 48 hours, active stimulation is rarely necessary. Should it become so, it should be carried out on broad general lines.

To ward off the second chill artificial heat, by hot water bags, etc., should be applied to patient, stimulating drugs, such as Tr. Capsicum and Chloric Ether should be given at first indication of chilliness, and, if necessary, inhalation of chloroform.

During convalescence, Sodium Phosphate should be given to keep the liver active; Iron, Strichnine and Arsenic as tonics; and at the first indication of jaundice moderately large doses of Calomel.

This, to the writer's best knowledge, is the general course adopted by the physicians of his locality for the treatment of this malady. That it is successful, he is thoroughly convinced, both from his own experience and from the fact that he almost daily sees persons who have been cured of from one to several attacks of malarial hemoglobinuria by its use, without a single dose of quinine. His experience of over six years in an intensely malarial section has taugh him what he believes to be several facts. Among these are, first, that while quinine is the specific for malaria, it is not always indispensable, as calomel will often abort and sometimes prevent a recurrence of malarial symptoms. Second, that in the black water regions, the physician must be extremely careful in the use of quinine during the fall months. Third, that the absorbed secretions of an abused and misunderstood liver, have an effect on the blood somewhat similar to that of the parasite itself; and fourth, that there are, as yet, many unsolved problems in malaria.

